# Photounbinding of Calmodulin from a Family of CaM Binding Peptides

**DOI:** 10.1371/journal.pone.0014050

**Published:** 2010-11-18

**Authors:** Klaus G. Neumüller, Kareem Elsayad, Johannes M. Reisecker, M. Neal Waxham, Katrin G. Heinze

**Affiliations:** 1 Department of Optical Engineering, Research Institute of Molecular Pathology, Vienna, Austria; 2 Department of Neurobiology and Anatomy, University of Texas Health Science Center, Houston, Texas, United States of America; University of Oulu, Germany

## Abstract

**Background:**

Recent studies have shown that fluorescently labeled antibodies can be dissociated from their antigen by illumination with laser light. The mechanism responsible for the photounbinding effect, however, remains elusive. Here, we give important insights into the mechanism of photounbinding and show that the effect is not restricted to antibody/antigen binding.

**Methodology/Principal Findings:**

We present studies of the photounbinding of labeled calmodulin (CaM) from a set of CaM-binding peptides with different affinities to CaM after one- and two-photon excitation. We found that the photounbinding effect becomes stronger with increasing binding affinity. Our observation that photounbinding can be influenced by using free radical scavengers, that it does not occur with either unlabeled protein or non-fluorescent quencher dyes, and that it becomes evident shortly *after* or *with* photobleaching suggest that photounbinding and photobleaching are closely linked.

**Conclusions/Significance:**

The experimental results exclude surface effects, or heating by laser irradiation as potential causes of photounbinding. Our data suggest that free radicals formed through photobleaching may cause a conformational change of the CaM which lowers their binding affinity with the peptide or its respective binding partner.

## Introduction

Fluorescent probes are commonly used in biological experiments and have provided enormous insight into cell machinery and protein dynamics. Despite their successful application over the last century, fluorescent conjugates can influence cell viability and the properties of the molecules under study [1] as well as the properties of a dye conjugated to a protein [2]. Particularly when using laser intensities beyond the fluorescence saturation limit, phototoxic reactions introduce major limitations in live cell fluorescence microscopy [3]. For techniques such as Fluorescence Recovery After Photobleaching (FRAP) or Fluorescence Loss in Photobleaching (FLIP), it has been shown that phototoxicity can be exerted not only on the illuminated cell but also on neighboring fluorescent cells [4]. Thus, understanding the photochemistry and photophysics of interactions between molecule and their conjugated labels is essential not only for avoiding pitfalls and data misinterpretations [5], but also for providing us with novel tools. Probes such as KillerRed [6] based on reactive oxygen species (ROS), techniques such as Chromophore-assisted light inactivation [7], or acceptor photobleaching [8] and saturation in FRET [9] show the great potential to capitalize on photophysical side-effects.

Recently it has been demonstrated that fluorescently labeled molecular complexes such as antibody-antigen [10] and toxin-receptor complexes [11] can be dissociated by light and rebind to the target. Unfortunately, this photo-induced phenomenon called “photounbinding” has been largely ignored and its basic mechanism is not yet understood. We believe that detailed knowledge of the processes involved would not only allow a systematic improvement of quantitative fluorescent studies, but also open the door for using photounbinding to induce or inhibit molecular interactions in a controlled fashion which may lead to the development of novel techniques and tools.

One important requirement for studying photounbinding is an assay that allows us to distinguish between the loss of a binding partner (photounbinding) from the loss of fluorescence by photobleaching. We have found that immobilizing one binding partner on a coverglass via a long chemical cross-linker [10] provides a solution. Vacant binding sites after photounbinding were visualized by subsequent rebinding of a differently labeled binding partner.

In the present photounbinding study, the emphasis was put on the dependence of the photounbinding phenomena on the initial dissociation constant of the molecular system under various experimental conditions in order to elucidate its underlying mechanism. To be able to perfom measurements using a single molecular system, we studied the binding of the signaling molecule calmodulin (CaM) to a family of peptides that mimic the CaM-binding domain of Ca^2+^/(CaM) dependent protein kinase II (CKII) [12]. These protein-peptide complexes exhibit different dissociation constants depending on the length of the CKII peptide. The synthetic peptides have been well characterized [12] and serve as an ideal model system to examine the dependence of photounbinding on binding affinity.

## Materials and Methods

### Mutagenesis, Expression, and Purification of CaM

The introduction of a single Cys residue by conversion of Asp at amino acid 3 to Cys in a pET23d CaM expression plasmid was described previously [13]. Note, that we term this construct CaM(C2) (and not CaM(C3) as originally described in [13]) as the initiating Met residue is removed from the protein when expressed in bacteria making the engineered Cys the second amino acid residue. Protein was produced by expression in the BL21(DE3)Star strain of *E.coli* (Invitrogen, San Diego, CA) and was purified as described previously [14] with minor modifications. Purified protein was dialyzed against 50 mM MOPS, pH 7.0, and stored at −20°C. The amount of CaM was quantified by a modified Bradford protein assay (Bio-Rad, Hercules, CA).

### Labeling of CaM(C2) with fluorescent dyes Alexa 647, Alexa 488, and quencher dyes QSY-9, ATTO540 Q

CaM labeling was performed as described previously [12] with minor modifications and precautions described in **supporting Material S1**. Labeled protein was dialyzed against 25 mM MOPS, pH 7.2, and stored at −20°C.

### Synthesis and purification of CKII peptides

All CKII peptides [12] listed in table 1 were synthesized with addition of an N-terminal Cys residue to allow for immobilization on the SM(PEG)_8_ crosslinker (Pierce).

**Table 1:** Summary of synthesized peptides used; K_d_ taken from [12].

Peptide	Sequence	K_d_×10^−13^ [*M*]
CKII(290–312)	CLKKFNARRKLKGAILTTMLATRN	3
CKII(292–312)	CKFNARRKLKGAILTTMLATRN	5
CKII(293–312)	CFNARRKLKGAILTTMLATRN	17
CKII(294–312)	CNARRKLKGAILTTMLATRN	570
CKII(290–312)*	LKKFNARRKLKGAILTTMLATRN	3

*: high affinity peptide used for unspecific background determination as described in **supporting Material S1**. This peptide cannot bind to the crosslinker (Assay CaM/CKII) due to the absence of an N-terminal Cys residue.

Synthesis was performed with assistance of the Protein Chemistry Facility of the Research Institute of Molecular Pathology, Vienna, Austria. The peptides were purified with High Performance Liquid Chromatography (HPLC) and verified by Mass Spectrometry.

### Staining for CaM/CKII and Immobilization Strategy

A selected CMKII peptide was covalently bound via a SM(PEG)_8_ crosslinker (MW 689.7) onto a coverslip by amino-silylation following the protocol recommended by Pierce (#80370, #22108), which is similar to the one described in Heinze 2009. The coverglasses were incubated with a 1 mM CKII peptide solution overnight at 4°C, rinsed thoroughly and incubated with CaM-A488, CaM-A647 (3 µM) or unlabeled CaM (60.4 µM) in buffer (25 mM MOPS, 150 mM KCL, 0.5 mM CaCl_2_, 0.1 mg/ml BSA) overnight at 4°C. Finally, the coated chamber was rinsed again and filled with 10 mL CaM-buffer. Proper coating was verified by fluorescence imaging.

When using peptides with lower binding affinity times, the periods between rinsing after re-incubation and imaging were kept short (less than 2 min) to minimize potential bias by spontaneous dissociation of the CaM-CKII peptide complex.

When using unlabeled CaM or the QSY 9 and Atto540 Q labeled CaM two different controls were performed to ensure the presence of the labeled nonfluorescent CaM and proper focusing onto the glass surface when inducing photounbinding. Details about the procedures and results are described in **supporting Materials S1**). For studying photounbinding in the presence of ascorbic acid as a chemical stablilizer, we used the dye A488 covalently bound to the SM(PEG)_8_ crosslinker by a tri-peptide (H-Gly-Gly-Cys-OH, #H-3325, Bachem, Germany) as an additional control.

### Staining with Phalloidin

For the Phalloidin staining, AAV-HT1080 cells (Stratagene, San Diego, CA, USA, #240109) were fixed in a 4% paraformaldehyde-PBS solution (PFA-PBS) for 15 min at RT, permeabilized with 0.1% Triton X-100 for 3 min, and blocked with 1% BSA-PBS solution for 30 min before incubation with phalloidin-Alexa488 (Ph-A488, Invitrogen, #A12379) for 60 min. After the photounbinding step cells were re-stained with phalloidin-Alexa647 (Ph-A647, Invitrogen, #A22287).

To test label-free unbinding the primary staining was done with unlabeled phalloidin and ph-A488 at a ratio of 4 (unlabeled):1 (labeled). A small amount of labeled phalloidin was necessary to visualize the actin filaments to be illuminated in the photounbinding step.

### Staining for Green Fluorescenct Protein (GFP)-actin

For GFP staining, PFA fixed B16 actin-GFP cells (kindly provided by the laboratory of Dr. Small, IMBA, Vienna, Austria) were permeabilized with 0.1% Triton X-100 and stained with anti-GFP-biotinylated/Streptavidin APC-Cy7 (BD Biosciences, San Jose, CA, USA, #554063). Cells were blocked in 1% BSA-PBS followed by incubation with goat anti-GFP (2.8 µg/mL) in PBS-BSA for 30 min each, washed (3×) with PBS and finally incubated with Streptavidin APC-Cy7 at the same concentration for 30 min at RT.

### Cell culture

For establishing B-16 actin-GFP mouse melanoma and AAV-HT1080 cultures, frozen cryovials were thawed in a 37°C water bath, transferred to 10 mL of DMEM (10% FCS, 2 mM L-Glutamine, Invitrogen), collected by centrifugation at 200×*g* for 3 min (RT), resuspended in 15 mL growth medium, and incubated at 37°C and 5% CO_2_. For passaging cells were washed with 10 mL prewarmed PBS, trypsinated (2 min, 5 mL trypsin-EDTA, Invitrogen, #25300) and resuspended in 5 mL DMEM. Finally, 1.5 mL of the cell suspension was transferred to 20 mL of DMEM in a flask. The cell density was monitored and maintained at 50% confluence.

### Photounbinding setup

For the unbinding experiments we used a laser scanning microscope (LSM) (Zeiss LSM 510 confocal) with options for one- and two-photon excitation (1PE or 2PE). To induce photobleaching and/or photo-unbinding the laser (488, 489, 543, 561 or 633 nm for 1PE or a modelocked Titan-sapphire laser line at 800 nm, 200 fs pulses, 80 MHz for 2PE) was focused onto the CaM/CKII peptide coated glass surface or the cell sample through the objective lens (Zeiss, Plan-Apochromat 63×/1.40 Oil DIC M27). Samples were raster scanned at 3.3 sec/line ( = 61µm/s) for 2PE and 62 msec/line ( = 3.25 mm/s) for 1PE, over a square subarea (edge length  = 10 or 20µm) - similar to the imaging procedures described in [10]. Imaging was performed in three detection channels (green [GFP, A488], yellow [A568], red [A647, APC-Cy7]). Excitation of green emitting dyes was provided by the 488 or 489 nm laser line, whereas excitation of Alexa568 (A568) by a 543 nm laser line, and Alexa647 (A647) and APC-Cy7 by a 633 nm laser line. For dual-color detection, fluorescence were selected using a LP505 (green) and a LP650 filter (red) emission filter.

### Data acquisition

The experimental procedures were equivalent to those described previously [10] in that they involved a four-step procedure: 1) Illumination of the protein-peptide complex to induce photounbinding (vacant binding sites); 2) Aquisition of a dual channel fluorescence image of the illuminated area. Green corresponds to GFP (assay GFP-actin) and CaM-A488 (assay CaM/CKII) and phalloidin-A488 (assay phalloidin), yellow to the IgG-A568 (CaM staining control, **supporting Material S1**) and red to the CaM-A647, (assay CaM/CKII), or Streptavidin APC-Cy7 (assay GFP-actin) or phalloidin-A647 (assay phalloidin); 3) Re-incubation with the same binding partner carrying a different fluorescent tag; 4) Aquisition of a second fluorescence image to quantify specific re-binding as a function of laser power. The laser power in 2) and 4) was always kept one order of magnitude below the fluorescence saturation limit to minimize additional unbinding.

### Computer-based data analysis

The fluorescence intensities in the green and red detection channel were obtained from surface plots of the CaM coated surface – CaM-A488 after bleaching and CaM-A647 after reincubation. The amount of rebinding (CaM-A647 fluorescence in the previously illuminated patches) and unbinding/bleaching (loss of CaM-A488 fluorescence) were calculated based on these surface plots. Raw data was analyzed using a custom-written computer code in the R-environment (see http://www.r-project.org/), which removed a linear background gradient. A more detailed disussion of the algorithm used is included in the **supporting Material S1**.

## Results

**Figure 1:**
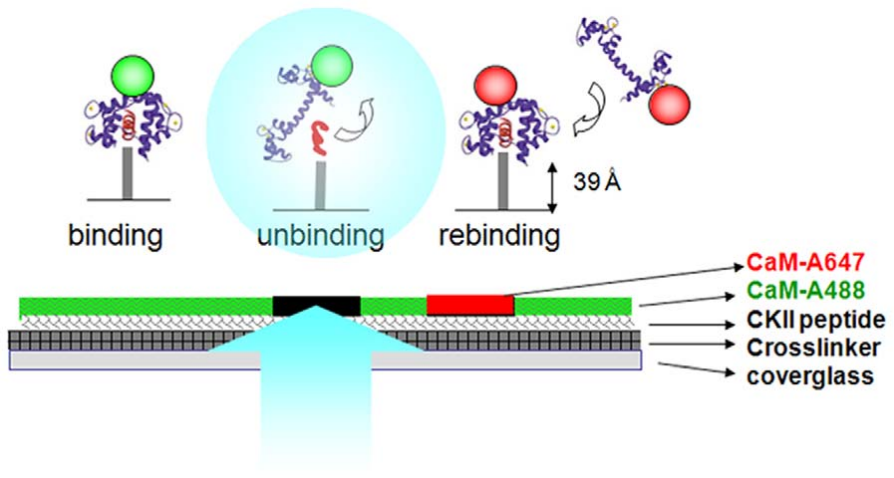
Schematic of the photounbinding assay and sample preparation. CKII peptides were attached to a glass surface via an SM(PEG)8 crosslinker followed by CaM-A488 incubation. After light illumination to induce photounbinding of the CKII peptide/CaM-488 complexes, the surface was re-incubated with CaM-A647 to visualize free binding sites in the previously illuminated regions.

To investigate how the binding of CaM to a set of CKII peptides is affected by photounbinding, we illuminated immobilized CaM/CKII peptide complexes with various laser intensities in a standard LSM and tested the photo-induced unbinding effect upon 1PE and 2PE on either fluorescent or non-fluorescent probes. One iteration of laser scanning was performed to induce photounbinding, unless stated otherwise. To assay photounbinding, we re-applied CaM – but with a different label – and quantified the fluorescence intensity of the newly bound probe. Figure 1 shows a sketched outline of the laser-induced unbinding setting. Several controls are described in the **supporting Material S1**.

### Fluorescently labeled calmodulin unbinds from a family of CaM binding (CKII) peptides

**Figure 2:**
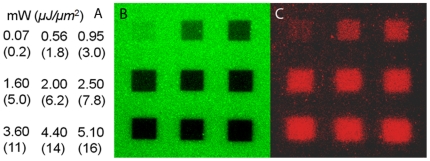
Unbinding of CaM-A488 and CKII(290-312) peptide by 488 nm laser light. A: laser power and intensity used to illuminate the corresponding patches in B: ‘bleaching’ pattern (CaM-A488 fluorescence, scale bar: 20 µm), and C: rebinding pattern (CaM-A647 fluorescence).

**Figure 3:**
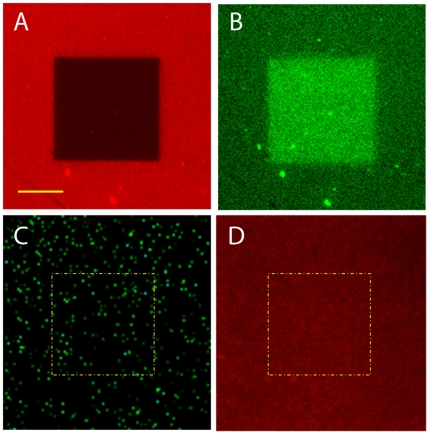
Photounbinding occurs for labeled, but not for unlabeled CaM. A: Illuminated patch of CaM-A647 and CKII(290–312) by 633 nm laser light; laser power: 190 µW (flux  = 589 nJ/µm^2^), scale bar  = 10 µm. B: rebinding pattern (CaM-A488 fluorescence). C: illumination of unlabeled CaM and CKII(290–312) peptide by 488 nm laser light within the indicated patch (yellow dashed line); laser power: 370 µW (flux  = 1.15 µJ/µm^2^), green dots: fluorescent beads to allow proper focusing. D: no rebinding of A647 was observed after laser illumination within the corresponding patch.

Laser illumination for inducing photobleaching and photounbinding of fluorescently tagged (Fig. 2B: A488; Fig. 3A: A647) or untagged (Fig. 3C) CaM was performed. To visualize unbinding the CaM/CKII(290–312) peptide coated coverglass chambers were incubated with the (counter)-tagged CaM (A647, A488) (Fig. 2C, Fig. 3D) followed by confocal dual-color imaging [channel 1: green (489 nm), and channel 2: red (633 nm)]. As shown in the images in Fig. 2B, square patches were scanned on the coverglass using 489 nm laser light at various powers, from 72 µW–5.4 mW or pulsed 800 nm laser light (16 mW–33 mW, data not shown). The coordinates of each intensity patch are shown in (Fig. 2A) or given in the figure caption of Fig. 3. To ensure that unlabeled CaM was within the focus during laser illumination sparsely distributed green fluorescent beads (diameter: 40 nm) were used to facilitate proper focusing onto the glass surface (Fig. 3C).

The confocal images in Fig. 2B demonstrates that laser illumination above 0.072 mW produced a loss of fluorescence in the CaM-A488 layer, which became stronger with increasing laser power. We note that laser intensities of <0.1 mW (inducing only a weak loss of fluorescence) already resulted in a clearly detectable CaM-A647 rebinding pattern (Fig. 2C). The rebinding of CaM-A647 (red patches in Fig. 2C) to the same areas after the laser exposure shows that the binding sites have (partly) become accessible to the new CaM-A647. Note, that exclusive photobleaching would simply result in a diffuse homogeneous fluorescence (i.e. background non-specific binding) after post-incubation with CaM-A647, and not in a strong correlation between the darkness of the patches in Fig. 2B and the brightness of the red fluorescence at the same patch locations in panel C as observed here.

Furthermore, we found that photounbinding of CaM requires a fluorescent label but is not restricted to a specific label or wavelength [10]. Figure 3 summarizes results of photounbinding of CaM-A647 (panel A,B), and unlabeled CaM (Panel C,D). While rebinding was observed for all labeled versions of CaM (also for identically labeled CaM - see **supporting Material S1**), no photounbinding and thus no rebinding was observed for unlabeled CaM after 1PE or 2PE at any laser intensity.

### Photounbinding is dependent on the initial dissociation constant of the CaM/CKII peptide complex

The calmodulin-CKII peptide system allows the study of photounbinding under different dissociation constants without changing the molecular system. Table 1 summarizes the dissociation constants of CaM and the CKII peptides used. The photounbinding performance of four CaM binding peptides with different binding affinities to calmodulin - spanning three orders of magnitude - were compared at various laser intensities.

Sample preparations and reactions with different CKII-peptides were performed in parallel under identical conditions (concentrations, incubation time, illumination and imaging settings) for each series of measurements. The CaM/CKII peptide coated surface was immersed in buffer at an initital temperature of 4°C to lower off-rates by 2–4 fold [12] and thereby minimize spontaneous CaM dissociation. To avoid overestimation of photounbinding, the decrease in the off-rates was conservatively assumed to be only two-fold. Additionally, rebinding values were mathematically corrected for the fluorescence loss due to CaM dissociation before the experiment has been finished. For analysis details see **supporting Material S1**.

**Figure 4:**
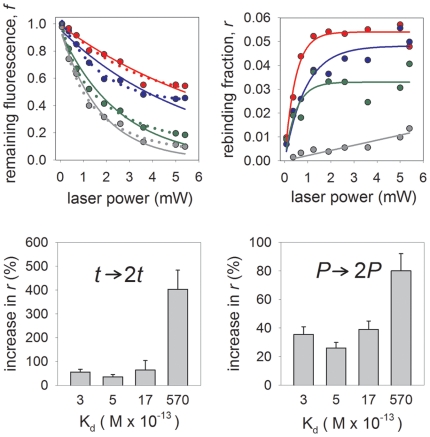
Photounbinding is dependent on the initial dissociation constant of the molecular system. Remaining Fluorescence (A) and corresponding rebinding (B) at various laser powers for peptides CKII(290–312) (grey symbols), CKII(292–312) (green symbols), CKII(293–312) (blue symbols), and CKII(294–312) (red symbols). A: single exponential (solid line) and double exponential (dotted line) fits to the unbinding data. B: single rising-exponential fits to the rebinding data. C: summary of maximal photounbinding values for all tested peptides after 1PE laser illumination (λ_exc_ = 488 nm; P = 3.6 mW) and one scan iteration (solid bars) and two scan iteration (open bars) in comparison. D: photounbinding threshold decreases for the lower affinity peptides (K_d_: 3–570×10^−13^ M) the graph shows the relative increase of rebinding when photounbinding laser power is doubled to 7.2 mW. Uncertainties for the rebinding fraction and remaining fluorescence fraction due to variablilty in CKII-CaM coatings and alignment of the coverglasses are less than 15% for each data point, whereas those associated with the laser power are negligible.

In Fig. 4A we plot the average remaining fluorescence (*f*) of CaM-A488 bound to different peptides after a single laser scan iteration as a function of the laser power; Fig. 4B shows the corresponding rebinding value *r* (measured after re-incubation with CaM-A647). The value *f* is in each case normalized such that *f = (f_p_−b_f_)/f_0_* where *f_p_* is the remaining fluorescence intensity within the illuminated patch, *b_f_* is the (typically small) background offset determined by imaging a fully bleached area next to the patches, and *f_0_* is the average fluorescence intensity measured for equally-sized areas above and below the patch. Further details regarding these calculations can be found in the **supporting Material S1**. The value of *r* has similarly been normalized *r = (r_p_−b_r_)/r_max_* where *r_p_* is the measured fluorescence intensity of the rebinding species, *b_r_* the background signal, and *r_max_* the fluorescence intensity when only the ‘rebinding’ species (e.g. CaM-A647 in complete absence of CaM-A488) is bound to the respective peptide under otherwise identical experimental conditions to the rebinding step. The background *b_r_* is an offset due mainly to unspecific binding of labeled CaM to the glass surface which was determined using a labeled CaM bound to a high affinity CKII(290–312)* peptide *without* a Cystein residue. This value was always <10% that of *r_p_* (for details see **supporting Material S1**).

We found that photounbinding (after 1PE, λ_exc_ = 488 nm) is higher for lower dissociation constants (corresponding to initially tighter binding). In Fig. 4A we fit a single exponential and a double exponential function (the latter with a constant offset) to the unbinding data. The former would correspond to a single path process whereas the latter to two paths [15]. The vertical axis shows the remaining fluorescence and the horizontal axis the laser power that was applied for a constant illumination time (which is proportional to the total incident energy). Whilst a (2-parameter) single-exponential [[*f*_ub_ = *f*_ub_^(0)^ + *f*_ub_^(1)^exp(-*P*/P_0_^(1)^)] describe our data well, a (5-parameter) double exponential [*f*_ub_ = *f*_ub_^(1)^exp(-*P*/P_0_^(1)^) + *f*_ub_^(2)^exp(-*P*/P_0_^(2)^)] describes our data significantly better, especially at the higher laser powers (fitting statistics presented in **supporting Material S1**). However the limited data points along with their associated uncertainty mean that we cannot entirely rule out either possiblity. A log-log plot (included in **supporting Material S1**) rules out a polynomial dependence of the binding fraction on the illumination power. In Fig. 4B we fit a rising exponential [*f*_rb_ = *f*_rb_^(0)^(1-exp(*P*/P_∞_)] to the rebinding data. We find reasonable agreement for peptides with the highest and lowest binding. Note that multi-exponential fits to the rebinding data would be redundant due to the limited statistics and large uncertainties.

Additionally, we found that for lower affinity complexes the intensity threshold for photounbinding is shifted to higher light doses when either doubling the scan iterations (Fig. 4C) or doubling the applied laser power for a single scan (Fig. 4D). Panel C of Fig. 4 shows the average photounbinding values (n = 4) of the four peptides for one and two laser scan iterations (solid and open bars) at a bleaching intensity of 3.6 mW. The lowest level of light induced unbinding was found with the CKII(294–312); the highest for the CKII(290–312) peptide. We found that photounbinding is ≈80% stronger for the low affinity CKII(294–312) peptide when two (instead of one) scan iterations are used, while photounbinding only increased by ≈35% for the high affinity peptide CKII(290–312). When doubling the laser power to 7.2 mW (panel D) instead of doubling the scan iterations, we see an even stronger effect on dissociataion with unbinding fractions up to 80%; however, the CKII peptide-CaM complexes with lower binding affinity are now the most affected by photo-induced unbinding. From this experiment we conclude that the risk of photounbinding strongly increases for the otherwise less affected lower affinity complexes when scan iterations are repeated and/or most drastically when the laser power is increased.

**Figure 5:**
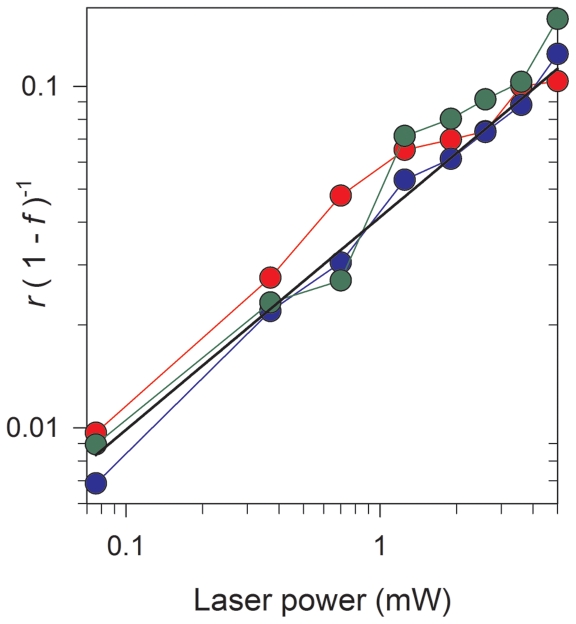
Plot of the rebinding to fluorescence-loss ration [*r*/(1−*f*)] as a function of laser power for a single line scan. Data is shown for peptides CKII(290–312) (grey symbols), CKII(292–312) (green symbols), CKII(293–312) (blue symbols), and CKII(294–312) (red symbols); The plot shows that the rebinding fraction is not directly proportional to the loss of fluorescence, but is suppressed at lower laser powers. The solid black line is a least-square fitted power-law to the CKII(293–312) peptide data (blue symbols) and given by: *r*/(1–*f*) = 0.04 *P* ^0.6^, where *P* is the laser power.

To understand the connection between photounbinding and photobleaching, we had a closer look at the relation between rebinding fraction *r* and the total decrease in fluorescence *f* for all peptides and found that they are not directly proportional (see Fig. 5). The Plot of the rebinding to fluorescence-loss ration [*r*/(1–*f*)] as a function of laser power suggest that the rebinding is suppressed at lower illumination energies (enhanced at higher energies). This thus suggests that unbinding is the result of a more elaborate underlying mechanism and not merely the byproduct of photobleaching (see further discussion below).

### Photounbinding of actin binding proteins in fixed cells

The cellular actin network and its interactions with various target proteins is one important topic in cell migration studies and is often addressed by fluorescence approaches [16]. The respective molecular assay is often realized using labeling of proteins by fusion to GFP family members or by using fluorescently labeled antibodies. We determined whether such a GFP-actin fusion protein in cells can be affected by photounbinding, and compared the results to an actin bound to phalloidin-A488.

We found that GFP cannot be dissociated from actin (for experimental details see **supporting Material S1**). However non-covalently bound fluorescent binding partners can in fact be dissociated from actin filaments as demonstrated by photounbinding of phalloidin-A488 from F-actin in fixed human fibrosarcoma cells. Phalloidin tightly binds actin subunits (K_d_ = 3.6×10^−8^ M as described previously in [17]) and stabilizes actin filaments [18].

**Figure 6:**
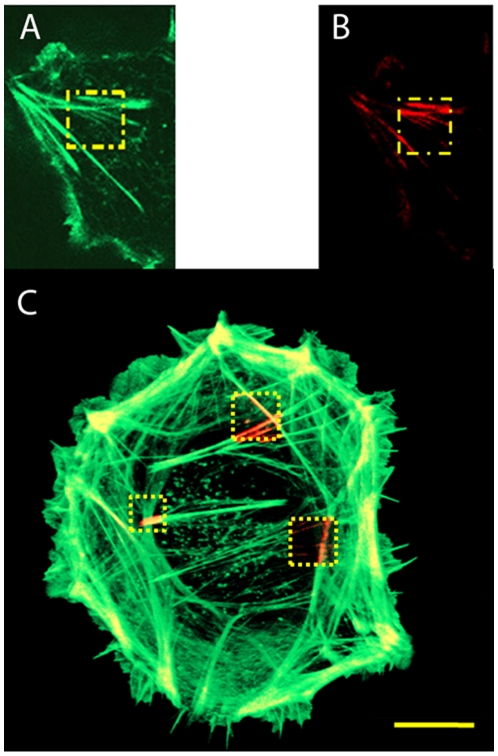
Photounbinding of labeled phalloidin from actin filaments. A: Ph-488 fluorescence after illumination at 488 nm (1PE) and 20 µW (62.0 nJ/µm^2^) (bleached patch is indicated in yellow); B: rebinding of Ph-647 within the previously illuminated area; C: photounbinding in a human fibrosarcoma cell, three squares were bleached (2PE, 800 nm) with different laser intensities left: 14 mW (flux  = 10.7 mJ/µm^2^); top: 20 mW (15.4 mJ/µm^2^); right: 24 mW (18.4 mJ/µm^2^).

Following labeling with phalloidin-A488, actin filaments were illuminated with different laser intensities (1PE: 488 nm, 20 µW–370 µW and 2PE: 800 nm, 14 mW–25 mW) and incubated with phalloidin-A647 directly after illumination. Fig. 6 shows clear phalloidin-A647 rebinding patterns (panel A,B 1PE at 20 µW, panel C, 2PE, at 14, 20 and 24 mW). Remarkably, a relatively low laser power of 20 µW (1PE) was already sufficient to photounbind phalloidin-A488 from actin filaments inside cells. As expected, photounbinding could not be observed for unlabeled phalloidin (data not shown). For the experiment, actin filaments were incubated with unlabeled and labeled phalloidin (for visualization) at a ratio of 4:1 and illuminated as described above. As a result, only a very slight increase of the phalloidin-A647 fluorescence in the bleached area was detected which can be explained by the small amount of labeled Phalloidin present. We thus can conclude that non-fluorescent phalloidin does not undergo photounbinding whereas fluorescently labeled phalloidin does.

### A radiative label is required for photounbinding

To further investigate whether a fluorescence label is the critical driving force to induce photounbinding we performed photounbinding experiments where CaM was labeled with a quencher dye, typically used in Fluorescence Resonance Energy Transfer (FRET) experiments as an ideal acceptor. The dyes QSY 9 and Atto540 Q used in this study exhibit a large cross-section at 560 nm and 542 nm, respectively, but very low fluorescence quantum efficiency. If the photounbinding mechanism relies on absorption, we should see CaM rebinding at the previously illuminated square patches. However, we did not observe photounbinding for any of the quencher dyes for any laser intensity applied in this study (**supporting Material S1**). First, this indicates that photounbinding requires a radiative label. Second, and most importantly, it indirectly suggests that photounbinding is not caused by laser-heating as heating depends on the absorption of the label and environment, which was comparable for both experiments using the fluorescent dye and the quencher dye label.

### Photounbinding is linked to photobleaching

Given the small ‘laser power window’ where photobleaching is observed without any signs of unbinding, we asked whether photounbinding and photobleaching follow independent mechanisms which occur simultaneously, or whether the two phenomena are linked.

**Figure 7:**
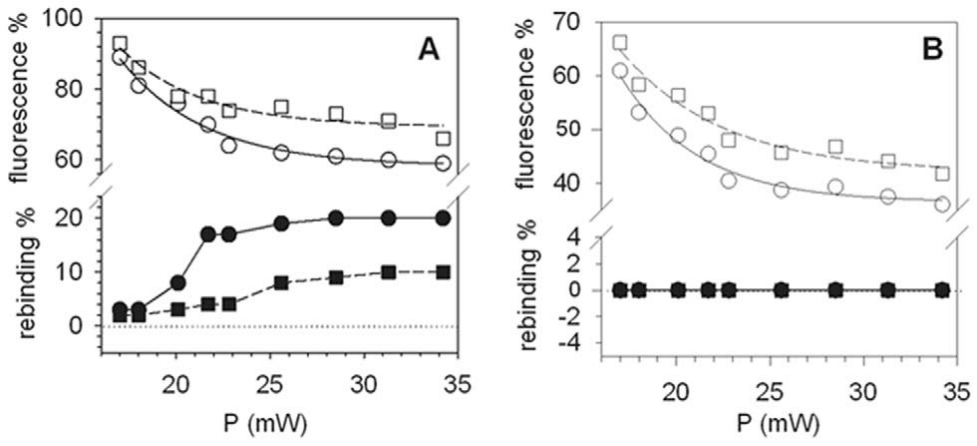
Photounbinding using the chemical fluorescence stabilizer ascorbic acid. A: Remaining CaM-A488 fluorescence (open symbols) and the corresponding rebinding (solid symbols) after two-photon excitation (Ti:Sa laser λ_exc_: 800 nm) with the addition of 8 mM ascorbic acid (squares) and without (circles). Photobleaching (and photounbinding) is partly prevented by the stabilizer as expected. B: Control study with A488 fluorophores directly covalently bound to the SM-PEG_8_ crosslinker via a tripeptide (H-Gly-Gly-Cys-OH). As expected the Alexa 488 fluorescence was stabilized to a comparable extent in presence of ascorbic acid (squares), however no photounbinding was detected. The two data sets have been fitted with a (2 parameter) single exponential function. Uncertainties for the rebinding fraction and remaining fluorescence fraction due to variablilty in CKII-CaM coatings and alignment of the coverglasses are less than 15% for each data point, whereas those associated with the laser power are negligible.

It has been described previously that, for the case of 2PE, preventing the bleaching pathway is possible using ascorbic acid as a chemical stabilizer (scavenger) [19]. If photounbinding and photobleaching are independent processes, then the fluorescence loss could not be (fully) prevented by a stabilizer as it prevents only photobleaching without altering the photounbinding fraction. However, if photounbinding always follows photobleaching we should observe a decrease in rebinding fraction with the stabilizer [19]. For the experiment shown in Fig. 7A, two identical CaM-488/CKII peptide samples in buffer were prepared, with one containing an addition of ascorbic acid during the photo-unbinding step at a concentration of 8 mM (pH adjusted to 7.2 by titration with HCl). After laser illumination of CaM-A488 (and re-incubation with CaM-A647) the buffer was replaced by PBS (without ascorbic acid).

Both samples (Fig. 7A) show a decrease in the CaM-A488 fluorescence after two-photon laser illumination (2PE, λ_exc._ = 800 nm, open symbols). However, in the presence of ascorbic acid the loss of fluorescence (Fig. 7A open squares) and the CaM-A647 rebinding (solid squares) were significantly smaller than for the sample without the scavenger (Fig. 7A open/solid circles). The stabilized fluorescence together with the decrease in rebinding in the presence of ascorbic acid suggests that free radicals known to be responsible for photobleaching after two-photon excitation [19] may also be responsible for the observed unbinding effect (details in the discussion section below). To ensure that this correlation is not an artefact, we performed a control study (Fig. 7B) using A488 fluorophores covalently bound to the SM-PEG_8_ crosslinker via a tripeptide (H-Gly-Gly-Cys-OH). As shown in Fig. 7B (open symbols) the Alexa 488 fluorescence in the presence of ascorbic acid was stabilized to a comparable extent to the CaM sample shown in Fig. 7A. However as expected for covalent bonds no photounbinding was detected (solid symbols). The two data sets in Fig. 7A and 7B show a comparable exponential decay and were fitted by a (2 parameter) single exponential function.

## Discussion

### Towards the unbinding mechanism

The suggested model is mainly based on three observations:

- Photounbinding increases with decreasing dissociation constant
- Unbinding (and rebinding) fractions are smaller in the presence of the reducing agent ascorbic acid (Fig. 7) and seem to follow the bleaching behavior of the labeled CaM but are not proportional.
- Non-radiative absorption is insufficient to induce photounbinding.

The increase in photounbinding with decreasing dissociation constant (increasing affinity), may be influenced by the unique conformational states that CaM adopts when complexed with these different peptides [20]. Since high affinity CKII peptides are stabilized by additional amino acid contacts with CaM [21], it is likely that the lowest energy state of the high affinity peptide-CaM complexes are mechanically more “rigid”. We speculate that this may in turn make them more susceptible to photounbinding, since one or more conformational changes in the CaM can be expected to make the bound (complex) state energetically less favourable. This is in contrast to the low affinity peptide where a larger number of conformational forms of CaM can be expected so the impact of photo-induced unbinding is more pronounced. If we assume that the unbinding is due to a conformational change in the CaM, there are several mechanisms that can in principle be responsible. Two of these are:

**1**) **Energy transfer from non-radiative relaxation in the fluorophore** [see **supporting Material S1** for some of the possible processes]. If photounbinding were driven by vibrational or other non-radiative relaxation transitions of the fluorophore, one would expect the photobleaching fraction and the rebinding fraction to have an opposite trend – i.e. increased photobleaching would in itself cause a decrease in the unbinding fraction. It also follows that the presence of a reducing agent in the solution should increase the total unbinding fraction.

**2**) **Photobleaching**. It has been shown in the past that ROS production can lead to oxidative damage and (reversible) conformational changes in proteins [22], [23]. Thus, it is likely that radicals produced by photobleaching can react with parts of the CaM or interfere with the CaM-peptide bonds. Here, one would expect a comparable trend between the photobleaching fraction and the unbinding fraction. Specifically, as the number of radical intermediate photobleaching products increases, the total number of interactions with the CaM capable of causing a conformational change should also increase. The exact dependence of the number of radical photobleaching products on the total incident flux will depend on the types of photobleaching events [24]–[30]. Unlike case 1), the presence of an appropriate reducing agent should always decrease the unbinding fraction.

Our results clearly show that the unbinding is decreased in the presence of the reducing agent ascorbic acid (Fig. 7). This suggests that a mechanism related to the formation of radicals [e.g. case 2)] plays an important role in the observed unbinding process. This is also in agreement with the observed positive correlation with the bleaching fraction (see Fig. 4A and 4B). Furthermore, the lack of photounbinding when CaM is labeled with quencher dyes suggests that the heat due to laser excitation is unlikely to cause the observed photounbinding.

The conformational change of the CaM itself may be assumed to be caused by its interaction with the resultant radicalized molecule X*. A subsequent reaction of a radical dark-state with, for example, free radicals in the solution eventually brings the fluorophore into a stable (bleached) non-fluorescent state.

The observation that the ratio *r*/(1−*f*) depends on laser power (Fig. 5) suggests that if photounbinding is a product of photobleaching then only a fraction of the pathways responsible for the bleaching will contribute.

**Figure 8:**
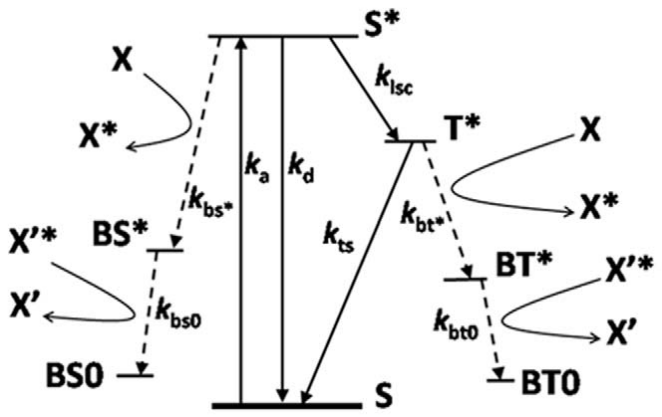
Jablonski energy diagram for the formation of radicals through bleaching of a generic flourophore (S). *k_a_*,: Excitation rate; *k_d_*: total (radiative & non-radiative) decay rate of the fluorophore from the excited- to the ground- singlet state; *k_isc_*: intersystem crossing rate; k_bs_* & k_bt_*: Bleaching rates of the excited singlet & triplet states into excited bleached (dark) states via radicalization of a surrounding molecule (X→X* or X'→X'*).

In Fig. 8 we show a simplified Jablonski energy diagram of possible decay mechanisms of a typical fluorophore. The diagram shows two known bleaching pathways from the excited singlet (S*) and the excited triplet (T*) states that eventually result in the stable non-fluorescent states BS0 and BT0. In each case a radical bleached intermediate (“dark”) state is formed (BS* & BT*) at the expense of a nearby molecule (X), which is radicalized. Whilst bleaching is often assumed to occur almost exclusively from the longer lived triplet excited state, it is also possible for the singlet excited state to also decay into radical dark states e.g. [30].

The observations that *r*/(1−*f*) increases with increasing laser power (Fig. 5), and the power dependence of the total bleaching fraction and rebinding fraction can be well described by a double- and single-exponential respectively (Fig. 's 4A & 4B), suggest that if the photobleaching indeed occurs via two separate pathways (viz. Fig. 8), then photounbinding is driven by the non-dominant path (the one with the smaller decay rate). Photounbinding which is directly related to bleaching via a second path with a slower decay rate than the dominant bleaching path would result in a value of *r*/(1−*f*) that increases with increasing laser power as shown in Fig. 5. This would likely correspond to the S*→BS*→BS0 path.

A reason why the excited singlet state bleaching may dominate for the photounbinding whereas excited triplet state bleaching does not, may be due to the higher energy provided by bleaching via the singlet excited state as compared to the triplet excited state. This speculation would also explain the larger photounbinding fractions observed for 2PE compared to 1PE, since the 2PE bleaching pathway is known to be significantly different for 2PE with no significant contribution coming from triplet state bleaching [19]. On the other hand the significant reduction in the observed photounbinding fraction in the presence of the radical scavenger ascorbic acid (which at the present time is understood to prevent only triplet state bleaching [19]) can be explained if the scavenger also reduces decay through the excited singlet state bleaching path. We emphasise that whilst the proposed explanation qualitatively explains our data, understanding the full complexities of the photounbinding will rely on having a better understanding of the energy landscape of the fluorophores in the studied system and the associated bleaching pathways and mechanisms.

### Conclusion and Outlook

Photounbinding has been shown to occur for various common binding systems such as antibody-antigen [10], protein-peptide [this work], as well as toxin-target interactions [11, this work]. It occurs in solution [10, this work], in cell culture [this work] as well as *in vivo* [11]. The maximum unbinding efficiencies per cycle of illumination were substantial and ranged between 20% [this work] and 85% [10] dependent on the molecular system under investigation and the excitation mode.

Photounbinding was visualized and quantified by rebinding the same, but differently labeled binding partners in the previously illuminated (photobleached) areas.

However, our results also suggest that photounbinding does not occur for molecules attached through covalent bonds. This is based on the two observations that there was no unbinding after crosslinking the binding partners by formaldehyde fixation (**supporting Material S1**), and no unbinding of A488 which was covalently linked to the SM-PEG_8_ crosslinker via a tripeptide. This hypothesis is further confirmed by a separate experiment probing GFP actin fusion proteins in cells, where GFP failed to be dissociated in GFP-actin fusion proteins.

For non-covalent binding of CKII peptides and A488-CaM we found that laser intensities of <100 µW −which induce only a weak loss of fluorescence− already result in a clearly detectable CaM-A647 rebinding pattern. Obviously, pure photounbinding is hard to distinguish from photobleaching followed by photounbinding of labeled CaM in a typical imaging setup at low or moderate laser intensities. This is particularly relevant in FRAP (Fluorescence Recovery after Photobleaching) or FLIP (Fluorescence Loss in Photobleaching) where experiments photounbinding could be misinterpreted as bleaching and bias the obtained results as discussed recently [10].

Our previous study [10] suggested that a fluorescence label is a requirement for photounbinding. Our results reinforce these findings as unlabeled CaM in solution and phalloidin in cells failed to be dissociated from their targets by light excitation (1PE at 488 nm and 2PE at 800 nm). Given that the quencher dyes used in this study did not produce unbinding, we can conclude that photounbinding is likely to be a radiative process requiring the emission of photons. Previously observed photounbinding when using a biotinylated secondary antibody tagged by a fluorophore labeled avidin [10] also strengthen this model. Assuming a radiative process the effective distance between molecule-fluorophore should indeed not make a difference for photounbinding, at least not at the distances relevant for fluorescence tagging. Follow up studies may focus on the process of absorption and emission or emission itself or by-products of the emission process as are known in the case of ROS production.

In this study we have found that the radical scavenger ascorbic acid prevents not only photobleaching, but also photounbinding under two-photon excitation. Our results suggest that the unbinding is either a direct consequence of photobleaching or at least follows similar pathways with similar thresholds. Whilst a reduction of photobleaching will reduce photounbinding, suggesting that photounbinding is related to a bleaching mechanism, the two are not proportional. The observed trend (increase in photounbinding fraction relative to bleaching fraction with increasing illumination energy) suggests that photounbinding may be governed solely by a sub-dominant bleaching pathway, such as that which occurs through the excited singlet state (S*).

Further experiments and theoretical work on the bleaching pathways of the chosen fluorophores would however be required to confirm this hypothesis.

A further possibility would be if unbinding were the result of a multi-photon process, where the fluorophore is excited into a higher singlet state, and photounbinding is the result of the subsequent decay. However this appears to be contradicted by the observation that doubling the time of illumination increases the photounbinding significantly more than doubling the laser intensity (Fig. 4C & D), and thus unlikely.

As the (CKII) peptides (dependent on their length) exhibit different dissociation constants for CaM, this system is ideally suited for learning more about photounbinding by studying its dependence on K_d_. It has been demonstrated that the four different CKII peptides selected (CKII(290, 292-, 293-, and 294–312)) show different rebinding levels to CaM-A647. With *increasing* dissociation constants of the CKII peptide/calmodulin complex, the photounbinding effect is *decreasing* and differed by a factor of ≈9 between the highest and lowest binding affinity peptides.

We have not yet fully understood, why the photounbinding rates increase with increasing binding affinity. It may be due to ligand-dependent CaM-oscillations [31], [32] or its rigidity. We cannot exclude that there is an (additional) distance-dependent effect − in the low affinity peptide the distance from the site of free radical formation could be greater, decreasing the probability that a dissociation reaction would occur. In contrast, with high affinity peptides, the fluorophore and subsequent free radical generated might find itself in closer proximity to the non-covalent bonds that are responsible for holding the complex together.

Future studies to elucidate the photounbinding mechanism would benefit from the use of single molecular fluorescence lifetime measurements in the presence of various reducing solutions to determine the dependence of the unbinding rate on the protein-peptide affinity. Molecular simulations of how these radicals interact with the CaM-peptide structure, and any conformational changes in the CaM they are able to induce, may provide us with further insights.

## Supporting Information

Material S1 Figure and text S1: Gel electrophoresis to test for monomeric CaM after quencher dye labeling. Figure and text S2: CaM antibody staining. Figure and text S3: Automated image analysis to quantify photounbinding. Text S4: Calculation of the total incident laser flux (incident energy per unit area). Figure and text S5: No bias by fluorescence quenching. Text S6: control: unspecific binding of CaM. Figure and text S7: Probing photounbinding by rebinding of identically labeled A488-CaM. Figure and table S8: Mathematical correction for low affinity peptides. Figure S9: Log-log plot of the unbinding and rebinding fraction. Figure and text S10: Analysis of photounbinding in fixed cells. Figure S11: No photounbinding for quencher dye labeled CaM. Figure S12: Non-radiative energy transfer to CKII-peptide (Jablonski energy diagrams). Text S13: Fitting statistics for plot. Figure S14: Photounbinding experiments before and after PFA fixation.
(1.22 MB PDF)

## References

[1] Bingaman S, Huxley VH, Rolando ER (2003). Fluorescent Dyes Modify Properties of Proteins Used in Microvascular Research.. Microcirculation.

[2] Luschtinetz F, Dosche C, Kumke MU (2009). Influence of Streptavidin on the Absorption and Fluorescence Properties of Cyanine Dyes.. Bioconjugate Chem.

[3] Hoebe RA, Van Der Voort HTM, Stap J, Van Noorden CJF, Manders EMM (2008). Qantitative Determination of the Reduction of Phototoxicity and Photobleaching by Controlled Light Exposure Microscopy.. J Microsc.

[4] Dobrucki JW, Feret D, Noatynsky A (2007). Scattering of Exciting Light by Live Cells in Fluorescence Confocal Imaging: Phototoxic Effects and Relevance for FRAP Studies.. Biophys J.

[5] Remington SJ (2006). Fluorescent Proteins: Maturation, Photochemistry and Photophysics. Curr.. Opinion in Struct Biol.

[6] Bulina ME, Chudakov DM, Britanova OV, Yanushevich YG, Staroverov DB (2006). A genetically encoded photosensitizer.. Nat Biotechnol.

[7] Bulina ME, Lukyanov KA, Britanova OV, Onichtchouk D, Lukyanov S (2006). Chromophore-assisted light inactivation (CALI) using the phototoxic fluorescent protein KillerRed.. Nat Protoc.

[8] Van Munster EB, Kremers GJ, Adjobo-Hermans MJ, Gadella TWJ (2005). Fluorescence resonance energy transfer (FRET) measurement by gradual acceptor photobleaching.. J Microsc.

[9] Beutler M, Makrogianneli K, Vermeij RJ, Keppler M, Ng T (2008). satFRET: estimation of Förster resonance energy transfer by acceptor saturation.. Eur Biophys J.

[10] Heinze KG, Costantino S, De Koninck P, Wiseman PW (2009). Beyond Photobleaching, Laser Illumination Unbinds Fluorescent Proteins.. J Phys Chem B.

[11] Akaaboune M, Grady MR, Turney S, Sanes JR, Lichtman JW (2002). Neurotransmitter Receptor Dynamics Studied In Vivo by Reversible Photo-Unbinding of Fluorescent Ligands.. Neuron.

[12] Waxham NM, Tsai A, Putkey A (1998). Mechansim for Calmodulin (CaM) Trapping by CaM-kinase II Defined by a Family of CaM-binding Peptides.. J Biol Chem.

[13] Kim SA, Heinze KG, Waxham MN, Schwille P (2004). Intracellular calmodulin availability accessed with two-photon cross-correlation.. Proc Natl Acad Sci USA.

[14] Gaertner TR, Putkey JA, Waxham MN (2004). RC3/Neurogranin and Ca^2+^/calmodulin-dependent protein kinase II produce opposing effects on the affinity of calmodulin for calcium.. J Biol Chem.

[15] Füreder-Kitzmüller E, Hesse J, Ebner A, Gruber HJ, Schütz GJ (2005). Non-exponential bleaching of single bioconjugated Cy5 molecules.. Chem Phys Lett.

[16] Parsons JT, Horwitz AR, Schwartz MA (2010). Cell adhesion: integrating cytoskeletal dynamics and cellular tension.. Nat Rev Mol Cell Biol.

[17] Faulstich H, Schäfer AJ, Weckauf M (1997). The Dissociation of the Phalloidin-Actin Complex.. Hoppe Seylers Z Physiol Chem.

[18] Barden JA, Miki M, Hambly BD, Dos Remedios CG (1987). Localisation of the Phalloidin and Nucleotide-Binding Sites on Actin.. Eur J Biochem.

[19] Dittrich PS, Schwille P (2001). Photobleaching and Stabilization of Fluorophores Used for Single-Molecule Analysis with One- and Two-Photon Excitation.. Appl Phys B.

[20] Meador WE, Means AR, Quiocho FA (1992). Target enzyme recognition by calmodulin: 2.4 A structure of a calmodulin-peptide complex.. Science.

[21] Junker JP, Ziegler F, Rief M (2008). Ligand-Dependent Equilibrium Fluctuations of Single Calmodulin Molecules.. Science.

[22] Hancock JT, Desikan R, Neill SJ (2001). Role of reactive oxygen species in cell signalling pathways.. Biochemical Society Transactions.

[23] Barcellos-Hoff MH, Dix TA (1996). Redox-mediated activation of latent transforming growth factor-beta 1.. Molecular Endocrinology.

[24] Lindqvist L (1960). A flash photolysis study of fluorescein.. Ark Kemi.

[25] Kasche V, Lindqvist L (1964). Reactions between the Triplet State of Fluorescein and Oxygen.. J Phys Chem.

[26] Usui Y, Itoh K, Koizumi M (1965). Switch-over of the Mechanism of the Primary Processes in the Photo-oxidation of Xanthene Dyes as Revealed by the Oxygen Consumption Experiments.. Bull Chem Soc.

[27] Koizumi M, Usui Y (1972). Fundamental aspects of the oxidative and reductive photobleaching of xanthene and thiazine dyes.. Mol Photochem.

[28] Hoogenboom JP, van Dijk EMHP, Hernando J, van Hulst NF, García-Parajó MF (2005). Power-Law-Distributed Dark States are the Main Pathway for Photobleaching of Single Organic Molecules.. Phys Rev Lett.

[29] Kuno M, Fromm DP, Johnson ST, Gallagher A, Nesbitt DJ (2003). Modeling distributed kinetics in isolated semiconductor quantum dots.. Phys Rev B.

[30] Yeow EKL, Melnikov SM, Bell TDM, De Schryver FC, Hofkens J (2006). Characterizing the Fluorescence Intermittency and Photobleaching Kinetics of Dye Molecules Immobilized on a Glass Surface.. J Phys Chem A.

[31] Putkey JA, Waxham NM (1996). A Peptide Model for Calmodulin Trapping by Calcium/Calmodulin-dependent Protein Kinase II.. J Biol Chem.

[32] Gsponer J, Christodoulou J, Cavalli A, Bui JM, Richter B (2008). A Coupled Equilibrium Shift Mechanism in Calmodulin-Mediated Signal Transduction.. Structure.

