# Supplementary material for: Photounbinding of Calmodulin from a Family of CaM Binding Peptides
Source: PLoS One. 2010 Nov 18;5(11):e14050. doi: 10.1371/journal.pone.0014050 (PMC2987815; doi:10.1371/journal.pone.0014050)
Supplement: Material S1 — Figure and text S1: Gel electrophoresis to test for monomeric CaM after quencher dye labeling. Figure and text S2: CaM antibody staining. Figure and text S3: Automated image analysis to quantify photounbinding. Text S4: Calculation of the total incident laser flux (incident energy per unit area). Figure and text S5: No bias by fluorescence quenching. Text S6: control: unspecific binding of CaM. Figure and text S7: Probing photounbinding by rebinding of identically labeled A488-CaM. Figure and table S8: Mathematical correction for low affinity peptides. Figure S9: Log-log plot of the unbinding and rebinding fraction. Figure and text S10: Analysis of photounbinding in fixed cells. Figure S11: No photounbinding for quencher dye labeled CaM. Figure S12: Non-radiative energy transfer to CKII-peptide (Jablonski energy diagrams). Text S13: Fitting statistics for plot. Figure S14: Photounbinding experiments before and after PFA fixation. (1.22 MB PDF) [file pone.0014050.s001.pdf]

Photounbinding of Calmodulin from a family of CaM binding peptides

Klaus G. Neumüller, [$] *Kareem Elsayad,* [$] *Johannes M. Reisecker,* [$] *M. Neal Waxham,* [†] *and Katrin G. Heinze* [$]

[$] Research Institute of Molecular Pathology, 1030 Vienna, Austria;

[†] Department of Neurobiology and Anatomy, University of Texas Health Science Center, Houston, Texas 77030

*corresponding author (Katrin.heinze@imp.ac.at)

Supporting material (figures and text):

Figure&text S1: Gel electrophoresis to test for monomeric CaM after quencher dye labeling

Figure&text S2: CaM antibody staining

Figure&text S3: Automated image analysis to quantify photounbinding

Text S4: Calculation of the total incident laser flux (incident energy per unit area)

Figure&text S5: No bias by fluorescence quenching

Text S6: control: unspecific binding of CaM

Figure&text S7: Probing photounbinding by rebinding of identically labeled A488-CaM

Figure&table S8: Mathematical correction for low affinity peptides

Figure S9: Log-log plot of the unbinding and rebinding fraction

Figure&text S10: Analysis of photounbinding in fixed cells

Figure S11: No photounbinding for quencher dye labeled CaM

Figure S12: Non-radiative energy transfer to CKII-peptide (Jablonski energy diagrams).

Text S13: Fitting statistics for plot.

Figure S14: Photounbinding experiments before and after PFA fixation

S1: Gel electrophoresis to test for monomeric CaM after quencher dye labeling

CaM(C2) has a tendency to dimerize via its Cys residue. To ensure its monomeric form 100 µM CaM in buffer (25 mM MOPS, 150 mM KCl, 0.5 mM $CaCl_2$, 0.1 mg/ml BSA, pH 7.2) was reduced with a 10-fold excess of dithiothreitol and heated to 60°C for 10 min and the result verified by SDS-PAGE (Fig. S1). For labeling with fluorescent dyes; Alexa 647 maleimide (Invitrogen, San Diego, CA, USA, #A20347) or Alexa 488 maleimide (Invitrogen, San Diego, CA, USA, #A10254) was dissolved in DMSO and added in 6.5-fold molar over both protein and dithiothreitol dropwise while stirring. The reaction was allowed to proceed overnight at 4ºC in the dark and was quenched using 10 mM β-mercaptoethanol. The labeled protein was purified using a Bio-Gel P6-DG (Bio-Rad, Hercules, CA)) column eluted with 50 mM HEPES, pH 7.0. Based on $\varepsilon_{\text{Alexa 633}}$ = 260,000/(M·cm) at 622 nm and $\varepsilon_{\text{Alexa 488}}$ = 77,000/(M·cm) at 493 nm, probe-to-protein ratios were determined to be ~0.9 for the labeled protein.

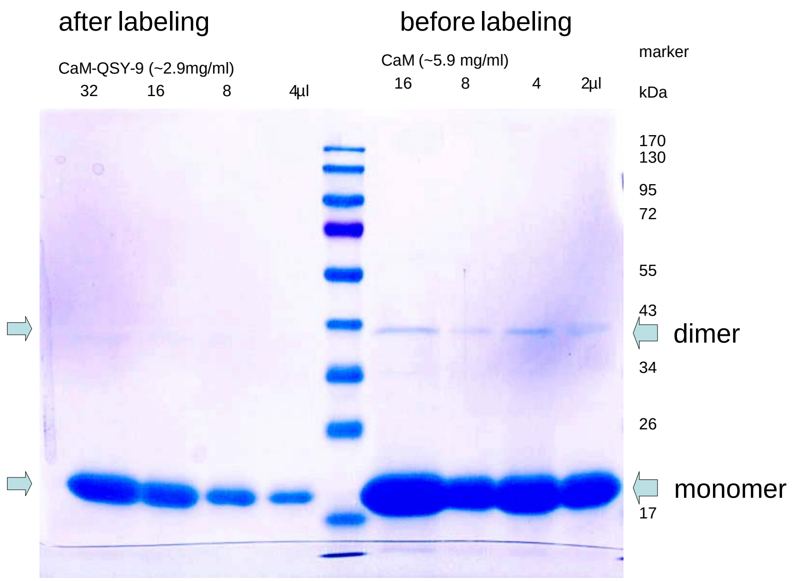

Figure S1: Polyacrylamide gel electrophoresis shows that CaM is present in its monomeric form after labeling with QSY-9. *Right: CaM before reduction with DTT showing monomer and dimer; Left: CaM after DTT reduction and labeling showing only monomer.*

For labeling with quencher dyes, dithiothreitol was first removed by exhaustive dialysis in 7 kDa molecular weight-cutoff Slide-A-Lyzer dialysis cassettes (Pierce, Rockford, IL, USA, # 66375).

Then labeling was accomplished by adding a 20-fold molar excess of either QSY-9 (Invitrogen, San Diego, CA, USA, #Q30457) or ATTO540 Q (Atto-tec, Siegen, Germany, #AD 540Q-41) dropwise to the stirred protein solution and the reaction was allowed to proceed for 2h at RT in darkness. Excessive reactive label was separated by extensive dialysis (~18 h) at 4°C.

These dyes show strong absorption at a specific wavelength range but no or very little fluorescence (Atto540 Q, abs. max.: 542 nm, QSY 9, abs. max.: 560 nm).

The required monomeric form of CaMC2) following labeling with QSY-9 was verified by a polyacrylamide gel (Fig. S1).

S2: CaM staining and fluorescent beads as a control in photounbinding experiments using unlabeled CaM

To ensure that the (invisible) unlabeled CaM was present on the glass surface and homogenously bound to the CKII(290-312) peptide coated cover-glass, we performed immunostaining using CaM Ab-4 (IgG), a mouse monoclonal antibody (Thermo Scientific, Fremont, CA, USA, MS-1268-P0) and ─ in the additional presence of the quencher dye ─ anti-mouse IgG–A568 or IgG-A488 (Invitrogen) respectively.

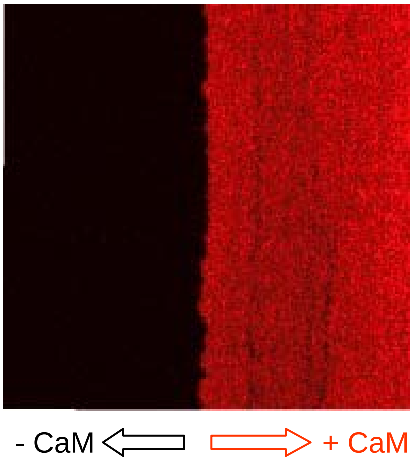

Figure S2: Staining of CaM/CKII coated surface with a primary anti-CaM (IgG) antibody and a secondary A568-anti-IgG. *Red fluorescence is specifically detected in the sample area (right) where CaM was previously applied.*

The CaM-antibody (primary antibody) was diluted in PBS to 1µg/mL and incubated on the CaM surface for 30 min at RT followed by rinsing the surface with CaM-buffer. Finally, anti-mouse IgG-A568 (secondary antibody) was incubated on the surface for 30 min at RT followed by thoroughly rinsing with CaM-buffer. The resulting surface was fluorescence imaged using an LSM (Zeiss, LSM 510 confocal). A fluorescent image of the stained calmodulin- peptide coated surface next to a CaM-free area was taken. The two areas (right: stained and left: unstained) in fig. S2 are clearly distinct and indicate the proper CaM coating of the experiment.

To ensure proper focusing, green fluorescent beads (diameter 0.04 µm, Invitrogen) were used in high dilution as an additive before incubating the glass surface with unlabeled CaM. The peptide coated cover-glasses were incubated with fluorescent highly diluted beads for several min and then rinsed with PBS. To avoid potential side effects to the photounbinding experiment, the dilution and incubation time was adjusted so that only few single beads were visible in the field of view of the LSM ─ just enough to adjust the focus plane. Finally, a CaM concentration of 60.4 µM was used for incubation of the peptide coated surface to guarantee complete saturation of all free binding sites.

S3: Computer-based data analysis

In this section we outline the details of the analysis we used to quantify photounbinding.

Firstly, the measured intensity of the line scans through the patches (lines 1, 2, and 3 in fig. 3a) were corrected for the background. The background intensity at each point on one of these lines was taken as the average of the intensity obtained from a line scan above and below the patch (i.e. lines-4 & -5 for the case of line-1, lines-5 & -6 for line 2, and lines-6 & -7 for line 3, see Fig. S3). The obtained background intensities at the same horizontal position were then subtracted from the corresponding intensities of lines 1-3. Since the background intensity was taken as the average of the intensity "above" the patch and "below" the patch, any linear gradient in the background (which would not be apparent from a single background measurement) is automatically accounted for. The illumination pattern is indicated in fig. S3. For lines 1, 2 and 3, corrected intensity values for an average of 30 pixels were calculated.

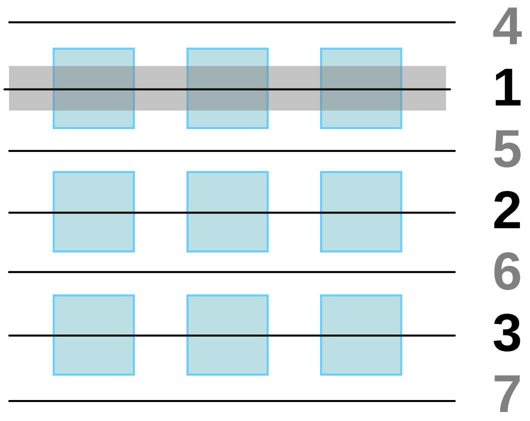

Figure S3: Schematic of the automated image analysis procedure used to quantify photounbinding. *The light blue patches indicate the areas of laser illumination. Typically each patch was illuminated with different laser intensity.*

Next data points that differed by more than two standard deviations from their immediate neighbors were excluded for further analysis and attributed to noise. Finally the results were

normalized to obtain estimates for the percentage rebinding and unbinding. Control measurements with "blocked" peptide coated coverglasses were performed and are discussed in the S2.

S4: Calculation of the total incident laser flux (incident energy per unit area)

To obtain the amount of energy deposited by the laser on a given area (the so-called "total incident flux") we perform a calculation identical to the one used previously (Heinze 2009). This calculation takes into account the finite scanning beam width (modeling the cross-sectional intensity profile as a Gaussian) which is large compared to the separation between parallel line scans[1]. This approximation can also be assumed for the experiments in the present study.
To keep this article as self contained as possible we quote the main equation below. For a more complete derivation as well as a quantitative discussion of the conditions of its validity we refer the reader to pp4-11 in the supplementary material of our previous work (Heinze 2009).

For both one and two photon excitation (1PE & 2PE) cases the average energy incident per unit area from the laser beam is calculated by:

$$\overline{E}_A = \frac{4\,P_0}{\pi w^2 v a}\left\{\int_0^{+\infty} dx \exp\left[-\left(\frac{x}{\sqrt{2}w}\right)^2\right]\right\}^2 .$$

Here P_0 represents the average laser power, a the distance between line-scans, $2w$ the effective beam width, and v the scan speed. For 1PE case P_0 is simply the total power, whereas for the 2PE case $P_0 = f\Delta t\, P_0^{(p)}$, where $P_0^{(p)}$ is the laser power associated with the pulses, f the pulse frequency, and Δt the pulse duration. For the case of our 2PE laser $f = 80$ MHz and $\Delta t = 200$ fs is given. The effective beam width, which is the same for the 1PE and 2PE cases, is given by $2w \approx 400$ nm, where as the distance between the line-scans is $a \approx 195$ nm. The effective scanning velocity is $v \approx$

[1] The condition for the validity of the derivation is significantly relaxed for the 2PE case, where it becomes: $v \ll w\,(1 - a/w)\,f$.

61 µm/s for the 2PE experiments, and $v \approx 3.25$ mm/s for the 1PE cases. In each of the figures in this manuscript the incident energy per unit area is quoted in parenthesis after the incident power.

S5: No bias by fluorescence label interactions of A488 and A647

Two fluorescent dyes in close proximity may show different emission and absorption characteristics than the same dyes when isolated, particularly due to fluorescent quenching. Fluorescence quenching refers to any process which decreases the fluorescence intensity of a given substance and can be induced by processes such as excited state reactions, Förster resonance energy transfer (FRET), complex-formation and others. Since a high amount of fluorescence loss by quenching could bias quantitative analysis of photounbinding we tested potential quenching effects between the used Alexa dyes (A488, A647). A CKII(290-312) coated cover-glass was incubated with equal concentrations of CaM-A488 and CaM-A647. Afterwards a square patch was laser illuminated with the blue (488 nm) or the red (633 nm) laser.

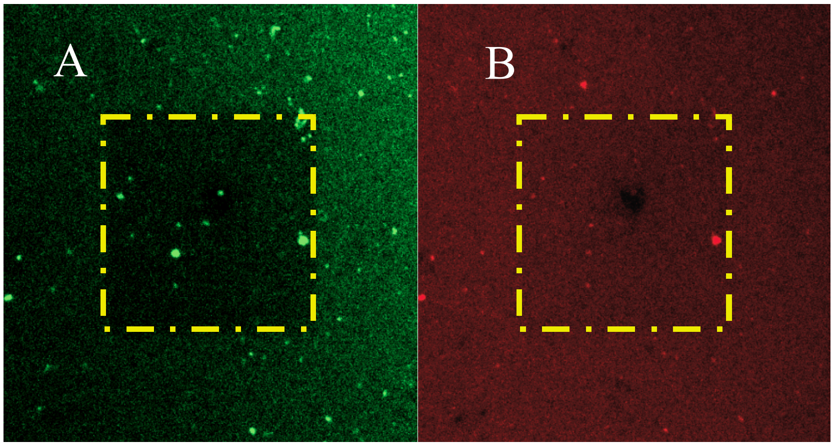

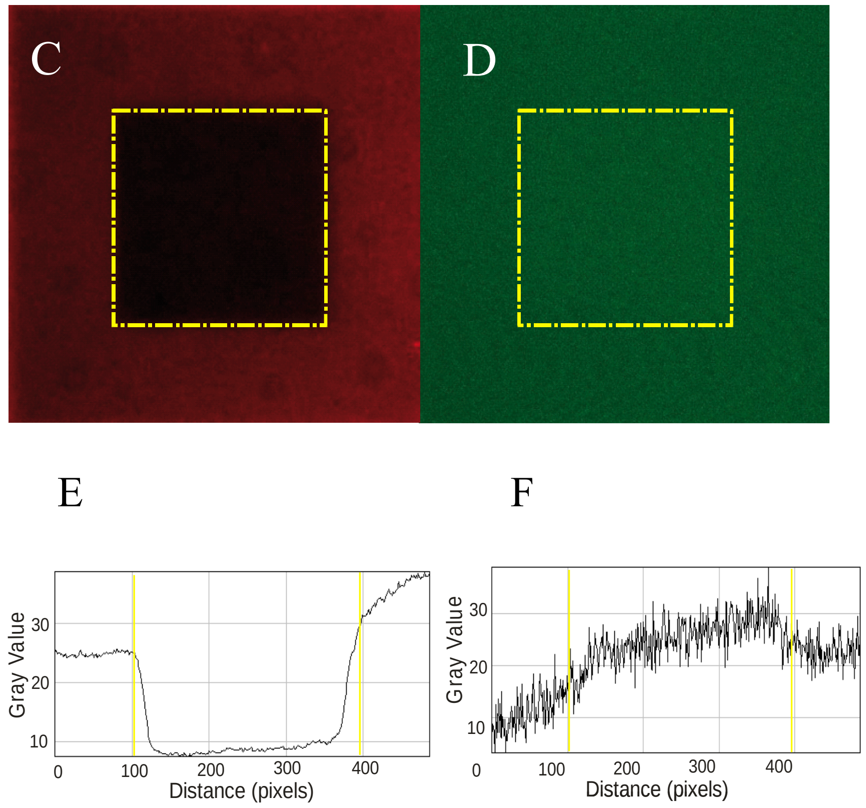

Figure S5: A CKII(290-312) coated cover-glass with equal concentrations of CaM-A488 and CaM-A647; *(A):CaM-A488 fluorescence after 488 nm excitation;(B) CaM-A647 fluorescence after 488 nm excitation; (C) CaM-A647 fluorescence after 633 nm excitation; (D) CaM-A488 fluorescence, after 633 nm excitation; (E, F) surface blot of C, D respectively.*

It was examined whether the CaM-A488 or CaM-A647 fluorescence increases, either when losing its potential quencher by bleaching or by unbinding after light excitation. As shown in fig. S5 CaM-A647 fluorescence (panel B) did not increase within the illuminated area when blue laser illumination (488 nm) was used to bleach the A488 counterpart (panel A). Thus, we assume that no quenching bias our results. As in our experiments, quantification of photounbinding was based on CaM-A647 rebinding and this figures shows that quenching has hardly any effect on this study. However it is worth noting that for bleaching at 633 nm (fig. S5 C,D), a slight increase (< 5%) of the CaM-A488 fluorescence was observed indicating that the A647 label (panel C, profile plot in E) quenches a small amount of the A488 fluorescence (panel D, profile in F). Thus, in a photounbinding experiment the remaining A488 fluorescence in the bleached square patches

may get slightly lowered by rebinding CaM-A647 possibly resulting in a slightly overestimated (~5%) unbinding values when switching dyes.

S6: control: unspecific binding of Calmodulin

The assay immobilized on a glass surface may imply the risk of unspecific binding of (labeled) CaM to the glass surface. This 'systematic background' has to be determined and controlled since such CaM background fluorescence would decrease the dynamic range of the experiments.

The amount of remaining unspecific CaM-A488 fluorescence and CaM-A647 rebinding in the presence of crosslinker, peptide was determined as follows: CKII peptides were attached to a cover-glass surface as described in *Material and Methods.* To minimize unspecific binding, the peptide-coated glass surface was blocked with a 2% BSA solution for 20 min as for every CaM/CKII photounbinding experiment. However, the binding site of CaM was blocked with a high affinity CKII(290-312)* peptide without a Cystein residue at the N-terminal end. Thus, this peptide could not bind to free SM(PEG)$_8$ crosslinkers on the cover-glass surface. CKII(290-312)* peptide incubation (20 mM) for several hours ensured that all CaM binding sites are saturated. The CaM concentration was equal to the concentrations that were used at all other photo-unbinding experiments (details see *Material and Methods*). After CKII coating, the surface was incubated with CaM-A488 and a square patch was illuminated onto the coated surface to be able to determine the unspecific binding and correct for any additional autofluorescence or light scattering background. Aditionally, we exposed CaM-A647 to the glass surface exhibiting no CaM-A647 rebinding pattern, which also indicates that the small amount of CaM-A647/CKII(290-312)* peptide attached to the surface unspecifically.

Due to the blocked binding site of CaM, the resulting A647 fluorescence on the glass surface reflects the amount of unspecific rebinding. For quantification we repeated the experiment described above with non-blocked CaM as a positive control and compared the respective average fluorescence intensities on the glass surface. The fraction of unspecific binding of labeled CaM was determined to approximately 9% using the software ImageJ and considered for all related photounbinding experiments.

S7: Rebinding of identically labeled Calmodulin

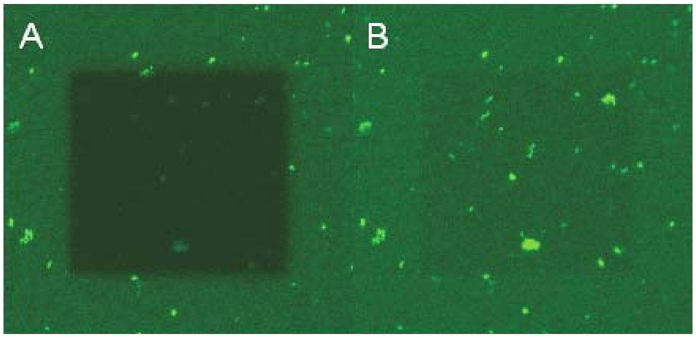

***Figure S7: Photounbinding assay with identically labeled A488-CaM**. (A): CaM-488 fluorescence after laser illumination (489 nm, 5.1 mW), and B: after rebinding with identically labeled CaM-488. The bright green spots in the image may be due to debris containing CaM-A488 which accidentally remained in the protein sample after insufficient centrifugation.*

A photounbinding experiment was performed with rebinding of an identically labeled CaM-A488. This control shows that the used CaM concentration in all reported experiments was sufficient to saturate the peptide coated surface and further demonstrate the universality of the observed effect. We found that fluorescence in the bleached area (s. fig. S7 A) was specifically restored (to a substantial part, S7 B): CaM-A488 rebound to free CKII peptides and fluorescence was recovered in the previously illuminated 'black' region. The ~6 % higher fluorescence intensity outside the area of interest after reincubation is most likely due to additional unspecific binding.

S8: Mathematical correction for low affinity peptides

The correction routine considered the amount of CaM that was lost from the peptide coated surface through diffusion. Our assumptions were based on the CaM-CKII off rates, previously measured by Waxham and colleagues (Waxham 1998) and allowed for determination of an error rate for the obtained rebinding intensities.

Particularly for the CKII(294-312) peptide (off rate: 0.002 s^{-1}) we found that the measured rebinding intensities were slightly underestimated (~ 20%). In contrast, the peptides CKII(290, 291, 292, 293 – 312) exhibiting smaller off rates were hardly affected by this aberration (error < 1.5 %).

All photounbinding values were corrected using the relation $k_{off} = -d[ln\ (P_N/P_{N0})]/dt$ (Baumgarth 2005), where k_{off} is the off rate, P_N the concentration of CaM/CKII peptide complex (CaM-CKII) at time t (here: 2 min), and P_{N0} the concentration of CaM-CKII at t=0. P_{N0} was set to 100% so that P_N could be obtained as the percentage of the remaining CaM-CKII. The corrected rebinding value is finally calculated by the measured rebinding value multiplied by $100/P_N$.

For a normalized $P_{N0}=1$ the table below shows the corrected fluorescent rebinding fractions (P_N) to be considered for the correction

PN	*t(sec)*	*off rates*	*CKII peptides*
0.787	120	0.002	294-312
0.988	120	0.0001	293-312
0.994	120	0.00005	292-312
0.996	120	0.000035	291-312
0.995	120	0.000045	290-312

Table S8: corrected fluorescent fractions (P_N)

S9: Log-log plot of the unbinding and rebinding fraction

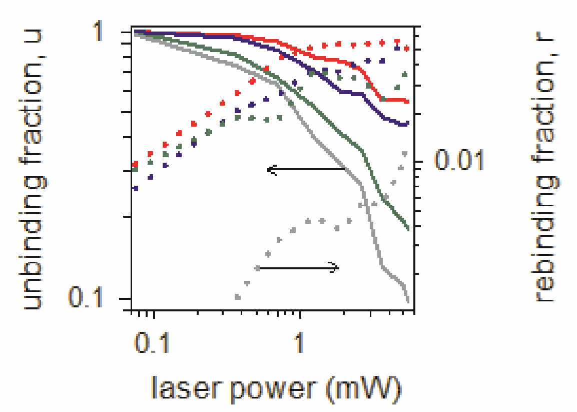

Fig. S9: Log-log plot of the unbinding and rebinding fraction *as a function of the illumination power, showing that a power-law dependence does not describe our data over a significant range of illumination powers.*

S10: Analysis of photounbinding in fixed cells

We found that in transgenic GFP-actin cells neither GFP can be dissociated from actin in transgenic GFP-actin filaments nor GFP-actin complexes can be dissociated from actin filaments in our photounbidning experiments. The GFP actin fusion protein is very stable as GFP is attached to actin via a peptide bond. The contacts between neighboring protomers in an actin dimer have been previously described and are mainly from hydrogen bonds, salt bridges and van der Waals contacts (Kudryashov 2005). It is known that such structures can be 'cut' by laser tools in so-called laser dissection routines (König 2001), however we would not expect to be able to selectively replace filaments with the moderate laser intensities used in our experiment. Whilst filaments may react in certain ways when illuminated by laser intensities that are slightly beyond photo-bleaching, the intensities used are not sufficient for laser dissection.

A small square area (10 μm^2) in a transgenic GFP-actin cell was laser illuminated (488 nm). Figure S10 shows the cell before and panel B after laser illumination. Finally, the cell was fixed and stained with anti-GFP-biotinylated/Streptavidin APC-Cy7 to test for rebinding. We found that the APC-Cy7 fluorescence (panel C, excitation 633 nm, emission: 650 nm, 750 nm) in the

previously illuminated area did not change, indicating that GFP could not be dissociated and actin filaments were not disrupted by light excitation.

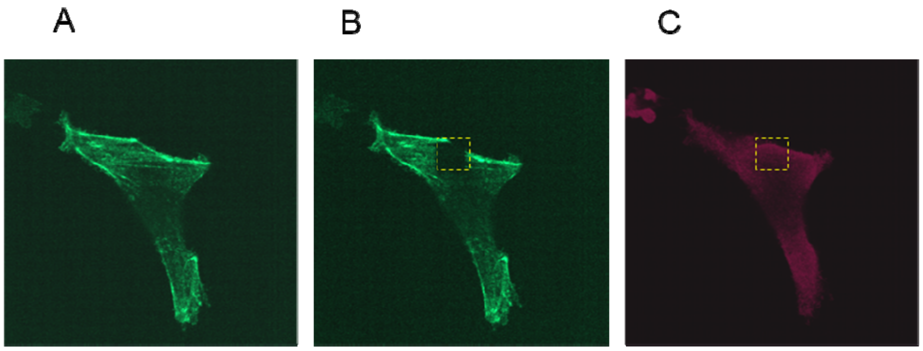

Figure S10: **No photounbinding of GFP-actin in B16 transgenic GFP-actin cells.** *A: GFP-actin fluorescence after illumination at 488 nm (1PE), 350 µW (1.09 µJ/µm^2) (bleached patch is indicated in yellow), (B) anti GFP staining; only background fluorescence, but no specific rebinding to the previously illuminated patch is observed.*

S11: No photounbinding for quencher dye labeled CaM

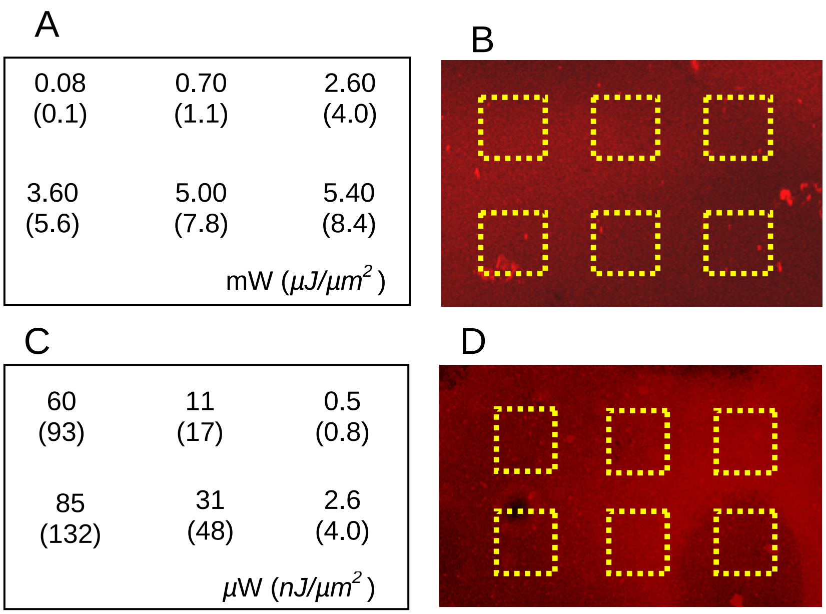

***Figure S11: No rebinding pattern when using non-fluorescent quencher dyes**. Square Patches as indicated in yellow were laser illuminated on the CaM-ATTO540 Q/peptide (B) and the CaM-QSY 9/peptide- and surface (D). The different laser intensities are indicated in the corresponding panels A and C. In both cases no rebinding pattern was observed. Fluorescent beads were used to ensure proper focusing during the bleaching and imaging steps (images not shown).*

S12: Non-radiative energy transfer to CKII-peptide (Jablonski energy diagrams)

non-

the

or

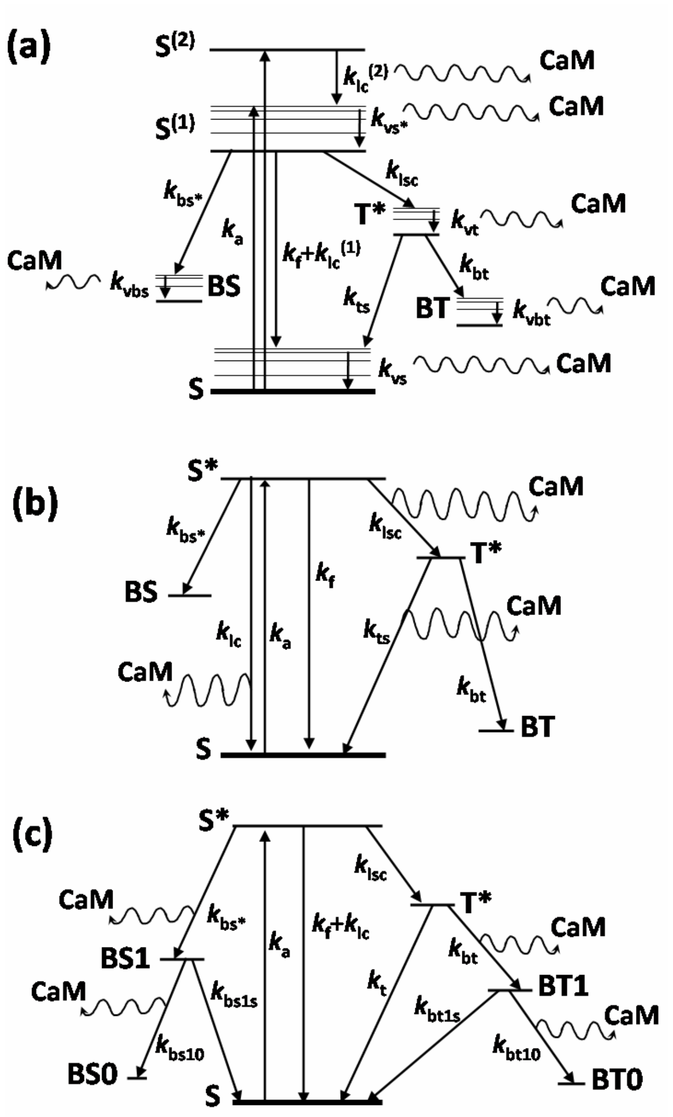

Figure S12: Non-radiative energy transfer to CKII-peptide

Jablonski energy diagram showing some radiative decay processes that may transfer energy to the peptide causing observed photounbinding [see case(1) in main text]. ***(a)*** *Energy is supplied by vibrational relatxation of excited states internal conversion between excited energy states;* ***(b)*** *energy is supplied by intersystem crossing or non radiativedecay to the singlet ground state;* ***(c)*** *energy is supplied by non-radiative decay to intermediate and/or stable bleached (dark) states.*

S13: Fitting statistics for plot in Fig. 3 (main text)

Single exponential (SE)

$f_{ub} = f_{ub}^{(1)}(0)\ \exp[-P/P_0^{(1)}]$ ……….unbinding and bleaching

$f_{rb} = f_{rb}(\infty)\ [1 - \exp(-P/P_0^{(1)})]$ ……….rebinding

Single exponential with off-set (SE+O)

$f_{ub} = f_{ub}^{(0)} + f_{ub}^{(1)}(0)\ \exp[-P/P_0^{(1)}]$ ……….unbinding and bleaching

$f_{rb} = f_{rb}^{(0)} + f_{rb}^{(1)}(\infty)\ [1 - \exp(-P/P_0^{(1)})]$ ……….rebinding

Double exponential (DE)

$f_{ub} = f_{ub}^{(1)}(0)\ \exp[-P/P_0^{(1)}] + f_{ub}^{(2)}(0)\ \exp[-P/P_0^{(2)}]$ ……….unbinding and bleaching

$f_{rb} = f_{rb}^{(1)}(\infty)\ [1 - \exp(-P/P_0^{(1)})] + f_{rb}^{(2)}(\infty)\ [1 - \exp(-P/P_0^{(2)})]$ ……….rebinding

Double exponential with offset (DE+O)

$f_{ub} = f_{ub}^{(0)} + f_{ub}^{(1)}(0)\ \exp[-P/P_0^{(1)}] + f_{ub}^{(2)}(0)\ \exp[-P/P_0^{(2)}]$ ……….unbinding and bleaching

---

Fig 3A

CKII(294-312) (red symbols)

(SE)

	Value(Standard Error)	Dependency
$f_{ub}^{(1)}(0)$	0.992(0.024)	0.4999
$P_0^{(1)}$	0.127(0.010)	0.4999
R^2 = 0.9564		

(SE+O)

	Value(Standard Error)	Dependency

$f_{ub}^{(0)}$	0.414(0.100)	0.9859
$f_{ub}^{(1)}(0)$	0.609(0.085)	0.9541
$P_0^{(1)}$	0.311(0.100)	0.9583

***R^2* = 0.9730**

(DE)

	**Value(Standard Error)**	**Dependency**
$f_{ub}^{(1)}(0)$	0.610(1.900)	0.9998
$P_0^{(1)}$	0.311(0.700)	0.9988
$f_{ub}^{(2)}(0)$	0.415(1.900)	1.0000
$P_0^{(2)}$	0.000(0.510)	1.0000

***R^2* = 0.9730**

(DE+O)

	**Value(Standard Error)**	**Dependency**
$f_{ub}^{(0)}$	0.415(0.380)	1.0000
$f_{ub}^{(1)}(0)$	0.360(0.150)	1.0000
$P_0^{(1)}$	0.310(0.030)	1.0000
$f_{ub}^{(2)}(0)$	0.294(0.150)	1.0000
$P_0^{(2)}$	0.311(0.026)	1.0000

***R^2* = 0.9730**

CKII(293-312) (blue symbols)

(SE)

	**Value(Standard Error)**	**Dependency**
$f_{ub}^{(1)}(0)$	0.960(0.040)	0. 4723
$P_0^{(1)}$	0.175(0.020)	0. 4723

***R^2* = 0.9239**

(SE+O)

	**Value(Standard Error)**	**Dependency**
$f_{ub}^{(0)}$	0.418(0.024)	0.8962
$f_{ub}^{(1)}(0)$	0.629(0.025)	0.6476
$P_0^{(1)}$	0.059(0.070)	0.8418

***R^2* = 0.9914**

(DE)

	**Value(Standard Error)**	**Dependency**
$f_{ub}^{(1)}(0)$	0.629(0.270)	0.9964
$P_0^{(1)}$	0.587(0.028)	0.9882
$f_{ub}^{(2)}(0)$	0.418(0.028)	0.9991
$P_0^{(2)}$	0.000(0.11)	0.9960

***R^2* = 0.9914**

(DE+O)

	**Value(Standard Error)**	**Dependency**

$f_{ub}^{(0)}$	0.418(0.090)	0.9892
$f_{ub}^{(1)}(0)$	0.323(>>1)	1.0000
$P_0^{(1)}$	0.310(>>1)	1.0000
$f_{ub}^{(2)}(0)$	0.294(>>1)	1.0000
$P_0^{(2)}$	0.311(>>1)	1.0000

***R^2* = 0.9914**

CKII(292-312) (green symbols)

(SE)

	Value(Standard Error)	Dependency
$f_{ub}^{(1)}(0)$	0.948(0.043)	0.4202
$P_0^{(1)}$	0.390(0.038)	0.4202

***R^2* = 0.9656**

(SE+O)

	Value(Standard Error)	Dependency
$f_{ub}^{(0)}$	0.175(0.022)	0.8491
$f_{ub}^{(1)}(0)$	0.844(0.027)	0.5732
$P_0^{(1)}$	0.690(0.064)	0.7981

***R^2* = 0.9939**

(DE)

	Value(Standard Error)	Dependency
$f_{ub}^{(1)}(0)$	0.457(0.070)	0.9594
$P_0^{(1)}$	1.523(0.340)	0.9313
$f_{ub}^{(2)}(0)$	0.604(0.076)	0.9895
$P_0^{(2)}$	0.230(0.032)	0.9449

***R^2* = 0.9981**

(DE+O)

	Value(Standard Error)	Dependency
$f_{ub}^{(0)}$	0.103(0.081)	0. 9952
$f_{ub}^{(1)}(0)$	0.305(0.210)	0. 9939
$P_0^{(1)}$	2.104(1.40)	0.9774
$f_{ub}^{(2)}(0)$	0.659(0.150)	0.9962
$P_0^{(2)}$	0.399(0.21)	0.9967

***R^2* = 0.9983**

CKII(290-312) (grey symbols)

(SE)

	Value(Standard Error)	Dependency

$f_{ub}^{(1)}(0)$	0.959(0.043)	0.4207
$P_0^{(1)}$	0.570(0.052)	0.4207

R^2 = 0.9764

(SE+O)

	Value(Standard Error)	Dependency
$f_{ub}^{(0)}$	0.098(0.025)	0.8013
$f_{ub}^{(1)}(0)$	0.909(0.035)	0.5308
$P_0^{(1)}$	0.796(0.084)	0.7597

R^2 = 0.9914

(DE)

	Value(Standard Error)	Dependency
$f_{ub}^{(1)}(0)$	0.529(0.210)	0.9841
$P_0^{(1)}$	1.470(0.650)	0.9550
$f_{ub}^{(2)}(0)$	0.512(0.225)	0.9948
$P_0^{(2)}$	0.319(0.110)	0.9592

R^2 = 0.9941

(DE+O)

	Value(Standard Error)	Dependency
$f_{ub}^{(0)}$	0.048(0.130)	0.9921
$f_{ub}^{(1)}(0)$	0.359(0.710)	0.9979
$P_0^{(1)}$	1.894(2.80)	0.9874
$f_{ub}^{(2)}(0)$	0.638(0.620)	0.9989
$P_0^{(2)}$	0.482(0.600)	0.9971

R^2 = 0.994

Fig 3B (rebinding) SE-fits

CKII(294-312) (red symbols)

	Value(Standard Error)	Dependency
$f_{rb}(\infty)$	0.054(0.001)	0.2705
$P_0^{(1)}$	2.167(0.270)	0.2705

R^2 = 0.96675

CKII(293-312) (blue symbols)

	Value(Standard Error)	Dependency
$f_{rb}(\infty)$	0.048(0.003)	0.4533
$P_0^{(1)}$	1.106(0.230)	0.4533

R^2 = 0.91204

CKII(292-312) (green symbols)

	**Value(Standard Error)**	**Dependency**
$f_{rb}(\infty)$	0.033(0.003)	0.2808
$P_0^{(1)}$	2.054(0.079)	0.2808
R^2 = 0.7058		

CKII(290-312) (grey symbols)

	**Value(Standard Error)**	**Dependency**
$f_{rb}(\infty)$	19.920(>>1)	1.0000
$P_0^{(1)}$	0.000(0.140)	1.0000
R^2 = 0.8427		

S14: Photounbinding experiments before and after PFA fixation

To investigate whether photounbinding is also able to break covalent bonds, we prepared two identical samples of immobilized A488-CaM/CKII peptide and performed PFA fixation of the protein-peptide surface *before* (sample 1) and *after* (sample 2) the bleaching/unbinding step. The surface was laser illuminated and reincubated with CaM-A647, to assess the rebinding. As shown in Fig. S13 A,B for case 1) no rebinding pattern (panel B) was observed meaning that the covalent linkage between CaM-A488 and the CKII peptides due to the PFA fixation remains intact. As shown in Fig. 4C,D for case 2) we observed a rebinding pattern (panel D) as the PFA fixation step did not crosslink the CKII(290-312)/CaM-A488 binding partners. The latter finding also serves as a control to ensure that the PFA chemistry does not interfere with the CaM-A647/CKII peptide rebinding.

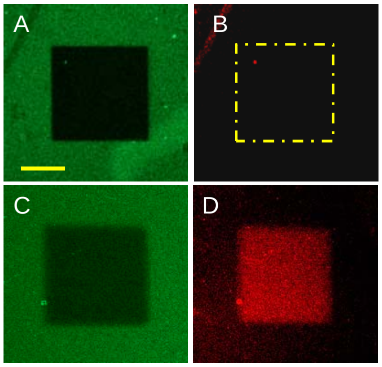

Figure S14: ***Photounbinding on a formaldehyde fixed CaM-A488/CKII(290-312) peptide****. Illumination at 800 nm [2PE] at 32 mW (flux = 24.6 mJ/µm^2), 2 iterations; A,B: crosslinking of the binding partners before the photounbinding step, A: 'bleaching' pattern (CaM-A488 fluorescence, scale bar: 10 µm) , B: no rebinding of CaM-A647 is observed within the previously illuminted area (yellow dashed patch); C,D: positive control: PFA fixation after reincubation (no crosslinking of the initial binding partners) leads to the typical rebinding pattern after incubation with CaM-A647.*

Reference:

Baumgarth B., F.W. Bartels, D. Anselmetti,A. Becker, and R. Ros. 2005. Detailed Studies of the Binding Mechanism of the Sinorhizobium Meliloti Transcriptional Activator ExpG to DNA. *Microbiology* **151**, 259-268.

König, K., I. Riemann, and W. Fritzsche. 2001, Nanodissection of Human Chromosomes with Near-Infrared Femtosecond Laser Pulses. *Opt. Lett.* **26**, 819-821.

Kudryashov, D.S., M.R. Sawaya, H. Adisetiyo, T. Norcross, H. György, E. Reisler, and T.O. Yeated. 2005. The Crystal Structure of a Cross-Linked Actin Dimer Suggests a Detailed Molecular Interface in F-Actin. *Proc. Natl. Acad. Sci. U.S.A.* **102**, 13105-13110.
